# Supplementary material for: Rhythmic Dynamics of Stress Granules in Wild-Type and Bmal1−/− Fibroblasts Lacking a Functional Canonical Circadian Clock
Source: Int J Mol Sci. 2025 Oct 13;26(20):9943. doi: 10.3390/ijms26209943 (PMC12564134; doi:10.3390/ijms26209943)
Supplement: Supplementary file 1 [file ijms-26-09943-s001.zip › ijms-3849482- Suppl Material revised.pdf]

Figure S1: Singular Spectrum Analysis of Temporal Oscillations in SG Number in NIH/3T3 Cells (related to Fig. 5)

(A) Singular values (spectrum)

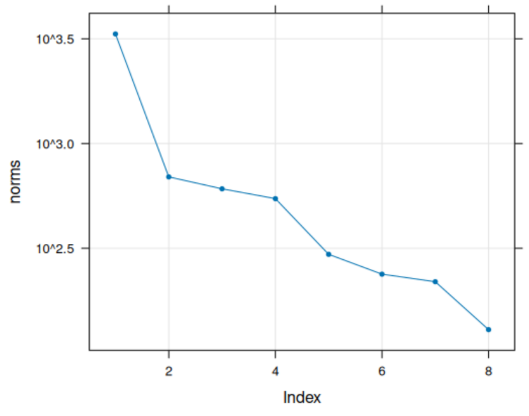

(B) First singular vectors

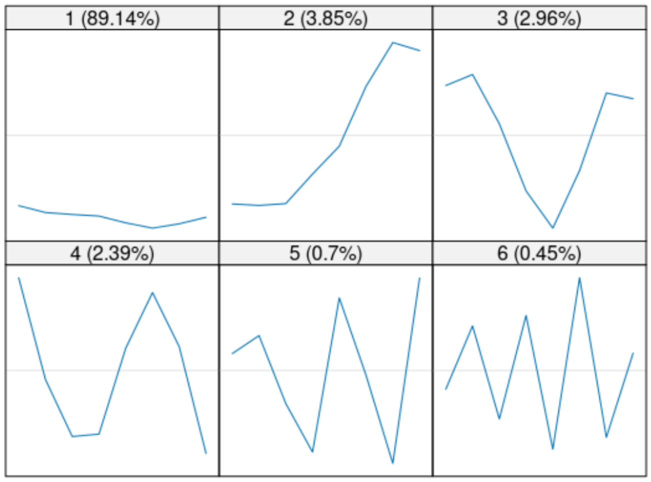

(C) Reconstructed cicadian rhythm

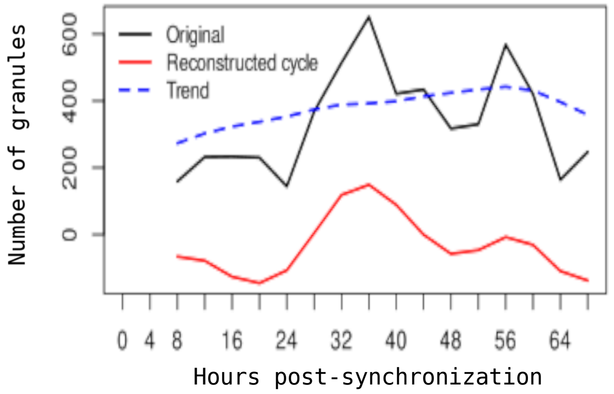

(D) Table of Estimated Periods

period	rate	Mod	Arg	Re	Im
35.560	0.023519	1.02380	0.18	1.00786	0.17996
-35.560	0.023519	1.02380	-0.18	1.00786	-0.17996
5.957	-0.011097	0.98896	1.05	0.48807	0.86014
-5.957	-0.011097	0.98896	-1.05	0.48807	-0.86014
2.179	-0.022117	0.97813	2.88	-0.94586	0.24916
-2.179	-0.022117	0.97813	-2.88	-0.94586	-0.24916

(A) Singular value spectrum of the time series of stress granule (SG) number. The first component (index 1) captures most of the variance. **(B)** First six singular vectors obtained from the SSA decomposition, with the proportion of variance explained indicated for each. Components 2 and 3 display oscillatory patterns consistent with circadian periodicity. **(C)** Reconstruction of the circadian rhythm in SG number. The original time series (black line) was decomposed into a trend component (blue dashed line) and a reconstructed rhythmic component (red line), revealing weak but detectable circadian oscillations. **(D)** Estimated periods derived from the spectral decomposition. The analysis identified periodicities around 35.6 h, ~6 h, and ~2.2 h. These components may reflect the mixture of trends, ultradian rhythms, and noise in the time series. Although a ~24 h periodicity is not explicitly captured in this table, the reconstructed signal in (C) suggests a weak circadian component.

Table S1 : Tukey HSD with Benjamini-Hochberg correction for relative *Bmal1* mRNA in NIH/3T3 cells (see Fig. 3)

Time (h)	7	14	21	28	35	42	49
**14**	1,000						
**21**	1,000	1,000					
**28**	0,997	1,000	0,350				
**35**	1,000	1,000	0,601	1,000			
**42**	1,000	1,000	1,000	0,857	1,000		
**49**	1,000	1,000	0,601	1,000	1,000	1,000	
**56**	0,997	1,000	0,350	1,000	1,000	0,857	1,000

***In bold p≤ 0.05**. Approximate Probabilities for Post Hoc Tests

Table S2: Tukey HSD with Benjamini-Hochberg correction for relative *Brf1* mRNA in NIH/3T3 cells (sewe Fig. 3)

Time (h)	7	14	21	28	35	42	49
**14**	1,000						
**21**	1,000	1,000					
**28**	0,997	1,000	0,350				
**35**	1,000	1,000	0,601	1,000			
**42**	1,000	1,000	1,000	0,857	1,000		
**49**	1,000	1,000	0,601	1,000	1,000	1,000	
**56**	0,997	1,000	0,350	1,000	1,000	0,857	1,000

***In bold p≤ 0.05**. Approximate Probabilities for Post Hoc Tests

Table S3 : Tukey HSD with Benjamini-Hochberg correction for relative *Lark1* mRNA in NIH/3T3 cells (see Fig. 3)

Time (h)	7	14	21	28	35	42	49
**14**	1,000						
**21**	0,442	1,000					
**28**	**0,001**	**0,000**	**0,000**				
**35**	0,933	0,147	**0,020**	**0,020**			
**42**	0,438	1,000	1,000	**0,000**	**0,020**		
**49**	1,000	1,000	0,707	**0,000**	0,635	0,701	
**56**	1,000	1,000	1,000	**0,000**	0,255	1,000	1,000

***In bold p≤ 0.05**. Approximate Probabilities for Post Hoc Tests

Table S4 : Tukey HSD with Benjamini-Hochberg correction for relative *Lark2* mRNA in NIH/3T3 cells (see Fig. 3)

Time (h)	7	14	21	28	35	42	49
**14**	**0,038**						
**21**	0,740	0,761					
**28**	0,207	**0,000**	**0,006**				
**35**	0,074	1,000	0,921	**0,000**			
**42**	0,749	0,749	1,000	**0,006**	0,912		
**49**	**0,005**	**0,000**	**0,000**	0,654	**0,000**	**0,000**	
**56**	0,069	1,000	0,912	**0,000**	1,000	0,912	**0,000**

***In bold p≤ 0.05**. Approximate Probabilities for Post Hoc Tests

Table S5 : Tukey HSD with Benjamini-Hochberg correction for relative *hnRNP Q1* mRNA in NIH/3T3 cells (see Fig. 3)

Time (h)	7	14	21	28	35	42	49
**14**	**0,000**						
**21**	**0,000**	1,000					
**28**	**0,000**	**0,001**	**0,001**				
**35**	**0,000**	0,742	0,715	**0,000**			
**42**	**0,000**	1,000	1,000	**0,001**	0,742		
**49**	**0,000**	**0,001**	**0,001**	1,000	**0,000**	**0,001**	
**56**	**0,000**	0,986	0,986	**0,000**	1,000	0,986	**0,000**

***In bold p≤ 0.05**. Approximate Probabilities for Post Hoc Tests

Table S6 : Tukey HSD with Benjamini-Hochberg correction for relative *hnRNP Q2* mRNA in NIH3T3 cells (see Fig. 3)

Time (h)	7	14	21	28	35	42	49
**14**	**0,000**						
**21**	**0,000**	1,000					
**28**	**0,000**	0,087	0,439				
**35**	**0,000**	1,000	0,411	**0,011**			
**42**	**0,000**	**0,041**	**0,007**	**0,000**	0,326		
**49**	**0,000**	1,000	1,000	0,087	1,000	**0,041**	
**56**	**0,000**	**0,036**	**0,006**	**0,000**	0,277	1,000	**0,036**

***In bold p≤ 0.05**. Approximate Probabilities for Post Hoc Tests

Table S7 : Tukey HSD with Benjamini-Hochberg correction for relative *TIA1* mRNA in NIH/3T3 cells (see Fig. 3)

**Time (h)**	**7**	**14**	**21**	**28**	**35**	**42**	**49**
**14**	0,329						
**21**	0,750	**0,017**					
**28**	0,392	**0,007**	0,999				
**35**	0,999	0,633	0,392	0,181			
**42**	0,999	0,054	0,999	0,999	0,750		
**49**	**0,014**	**0,000**	0,257	0,540	**0,007**	0,097	
**56**	0,999	0,969	0,207	0,079	0,999	0,450	**0,004**

***In bold p≤ 0.05**. Approximate Probabilities for Post Hoc Tests

Table S8: Tukey HSD with Benjamini -Hochberg correction for SG number per image in NIH/3T3 cells (see Fig. 5, Table 1)

**Time (h)**	**8**	**12**	**16**	**20**	**24**	**28**	**32**	**36**	**40**	**44**	**48**	**52**	**56**	**60**	**64**
**12**	1,000														
**16**	1,000	1,000													
**20**	1,000	1,000	1,000												
**24**	1,000	0,695	0,664	0,715											
**28**	**0,000**	**0,020**	**0,022**	**0,017**	**0,000**										
**32**	**0,000**	**0,000**	**0,000**	**0,000**	**0,000**	**0,007**									
**36**	**0,000**	**0,000**	**0,000**	**0,000**	**0,000**	**0,000**	**0,015**								
**40**	**0,000**	**0,000**	**0,000**	**0,000**	**0,000**	1,000	0,512	**0,000**							
**44**	**0,000**	**0,000**	**0,000**	**0,000**	**0,000**	1,000	0,764	**0,000**	1,000						
**48**	**0,005**	0,715	0,741	0,695	**0,001**	1,000	**0,000**	**0,000**	0,345	0,118					
**52**	**0,001**	0,442	0,475	0,402	**0,000**	1,000	**0,000**	**0,000**	0,649	0,312	1,000				
**56**	**0,000**	**0,000**	**0,000**	**0,000**	**0,000**	**0,000**	1,000	0,715	**0,006**	**0,020**	**0,000**	**0,000**			
**60**	**0,000**	**0,000**	**0,000**	**0,000**	**0,000**	1,000	0,491	**0,000**	1,000	1,000	0,363	0,664	**0,006**		
**64**	1,000	1,000	1,000	1,000	1,000	**0,000**	**0,000**	**0,000**	**0,000**	**0,000**	**0,005**	**0,001**	**0,000**	**0,000**	
**68**	0,695	1,000	1,000	1,000	0,326	0,067	**0,000**	**0,000**	**0,000**	**0,000**	1,000	0,723	**0,000**	**0,000**	0,715

***In bold p≤ 0.05**. Approximate Probabilities for Post Hoc Tests

Table S9: Tukey HSD with Benjamini -Hochberg for signal Intensity in NIH/3T3 cells (see Fig. 5, Table 1)

Time (h)	8	12	16	20	24	28	32	36	40	44	48	52	56	60	64
**12**	**0,005**														
**16**	0,197	1,000													
**20**	0,764	1,000	1,000												
**24**	0,976	0,976	1,000	1,000											
**28**	**0,000**	1,000	0,194	**0,020**	**0,011**										
**32**	**0,000**	**0,000**	**0,000**	**0,000**	**0,000**	**0,014**									
**36**	**0,000**	**0,000**	**0,000**	**0,000**	**0,000**	**0,000**	1,000								
**40**	**0,000**	**0,000**	**0,000**	**0,000**	**0,000**	**0,000**	1,000	1,000							
**44**	**0,000**	**0,000**	**0,000**	**0,000**	**0,000**	**0,032**	1,000	1,000	1,000						
**48**	**0,000**	0,386	0,014	**0,001**	**0,000**	1,000	0,248	**0,002**	**0,008**	0,428					
**52**	**0,000**	**0,000**	**0,000**	**0,000**	**0,000**	0,236	1,000	0,802	1,000	1,000	1,000				
**56**	**0,000**	**0,000**	**0,000**	**0,000**	**0,000**	**0,000**	0,151	1,000	1,000	0,050	**0,000**	**0,011**			
**60**	**0,000**	**0,000**	**0,000**	**0,000**	**0,000**	**0,000**	1,000	1,000	1,000	1,000	**0,011**	1,000	0,975		
**64**	**0,000**	1,000	0,972	0,298	0,199	1,000	**0,000**	**0,000**	**0,000**	**0,001**	1,000	**0,014**	**0,000**	**0,000**	
**68**	**0,000**	1,000	0,972	0,297	0,197	1,000	**0,000**	**0,000**	**0,000**	**0,001**	1,000	**0,011**	**0,000**	**0,000**	1,000

***In bold p≤ 0.05**. Approximate Probabilities for Post Hoc Tests

Table S10: Tukey HSD with Benjamini-Hochberg correction for SG area in NIH/3T3 cells (see Fig. 5, Table 1)

Time (h)	8	12	16	20	24	28	32	36	40	44	48	52	56	60	64
**12**	0,741														
**16**	1,000	1,000													
**20**	1,000	1,000	1,000												
**24**	1,000	1,000	1,000	1,000											
**28**	1,000	1,000	1,000	1,000	1,000										
**32**	0,410	1,000	1,000	1,000	1,000	1,000									
**36**	1,000	1,000	1,000	1,000	1,000	1,000	1,000								
**40**	1,000	0,124	0,395	0,270	1,000	1,000	0,055	1,000							
**44**	1,000	1,000	1,000	1,000	1,000	1,000	0,724	1,000	1,000						
**48**	1,000	**0,001**	**0,006**	**0,003**	0,065	0,175	**0,000**	0,248	1,000	0,784					
**52**	1,000	**0,017**	**0,073**	**0,049**	0,440	0,907	**0,006**	1,000	1,000	1,000	1,000				
**56**	1,000	0,123	0,392	0,268	1,000	1,000	0,055	1,000	1,000	1,000	1,000	1,000			
**60**	1,000	**0,047**	0,163	0,107	0,798	1,000	**0,017**	1,000	1,000	1,000	1,000	1,000	1,000		
**64**	**0,037**	**0,000**	**0,000**	**0,000**	**0,000**	**0,000**	**0,000**	**0,000**	0,160	**0,004**	1,000	0,813	0,162	0,390	
**68**	0,907	**0,000**	**0,001**	**0,000**	**0,016**	0,053	**0,000**	**0,070**	1,000	0,316	1,000	1,000	1,000	1,000	1,000

***In bold p≤ 0.05**. Approximate Probabilities for Post Hoc Tests

Table S11: Tukey HSD with Benjamini-Hochberg correction SG number per image in *wt* cells (see Fig. 6, Table 1)

Time (h)	7	14	21	28	35	42	49
**14**	1,000						
**21**	1,000	1,000					
**28**	0,815	0,467	0,199				
**35**	**0,000**	**0,000**	**0,000**	**0,047**			
**42**	**0,000**	**0,000**	**0,000**	0,184	1,000		
**49**	0,893	1,000	1,000	**0,012**	**0,000**	**0,000**	
**56**	0,074	**0,018**	**0,005**	1,000	0,640	1,000	**0,000**

***In bold p≤ 0.05**. Approximate Probabilities for Post Hoc Tests

Table S12: Tukey HSD with Benjamini-Hochberg correction signal intensity in *wt* cells (see Fig. 6, Table 1)

Time (h)	7	14	21	28	35	42	49
**14**	1,000						
**21**	1,000	1,000					
**28**	**0,000**	0,000	**0,000**				
**35**	**0,000**	**0,000**	**0,000**	1,000			
**42**	**0,000**	**0,000**	**0,000**	**0,001**	0,096		
**49**	1,000	1,000	1,000	**0,000**	**0,000**	**0,000**	
**56**	0,077	0,052	**0,001**	**0,000**	**0,000**	**0,003**	0,109

***In bold p≤ 0.05**. Approximate Probabilities for Post Hoc Tests

Table S13: Tukey HSD with Benjamini-Hochberg correctionSG area in *wt* cells (see Fig. 6, Table 1)

Time (h)	7	14	21	28	35	42	49
**14**	1,000						
**21**	1,000	1,000					
**28**	**0,000**	**0,000**	**0,000**				
**35**	**0,000**	**0,000**	**0,000**	0,500			
**42**	**0,000**	**0,000**	**0,000**	**0,035**	1,000		
**49**	1,000	1,000	1,000	**0,000**	**0,000**	**0,001**	
**56**	**0,008**	**0,001**	**0,039**	**0,000**	0,276	1,000	0,050

***In bold p≤ 0.05**. Approximate Probabilities for Post Hoc Tests

Table S14: Tukey HSD with Benjamini-Hochberg correction for SG number per image in *Bmal1 -/-* cells (see Fig. 6, Table 1)

Time (h)	7	14	21	28	35	42	49
**14**	0,135						
**21**	**0,000**	0,592					
**28**	1,000	0,879	**0,013**				
**35**	**0,001**	**0,000**	**0,000**	**0,000**			
**42**	**0,000**	**0,000**	**0,000**	**0,000**	1,000		
**49**	0,317	**0,000**	**0,000**	**0,013**	0,592	0,146	
**56**	**0,000**	**0,000**	**0,000**	**0,000**	1,000	1,000	0,135

***In bold p≤ 0.05**. Approximate Probabilities for Post Hoc Tests

Table S15: Tukey HSD with Benjamini-Hochberg correction signal intensity in *Bmal1 -/-* cells (see Fig.6, Table 1)

Time (h)	7	14	21	28	35	42	49
**14**	**0,000**						
**21**	**0,000**	0,984					
**28**	**0,001**	**0,000**	**0,000**				
**35**	**0,000**	**0,000**	**0,000**	0,097			
**42**	**0,009**	**0,000**	**0,000**	1,000	0,051		
**49**	0,976	**0,000**	**0,000**	0,115	**0,000**	0,331	
**56**	**0,006**	**0,000**	**0,000**	1,000	**0,019**	1,000	0,326

***In bold p≤ 0.05**. Approximate Probabilities for Post Hoc Tests

Table S16: Tukey HSD with Benjamini-Hochberg correction SG area in *Bmal1 -/-* cells (see Fig.6, Table 1)

Time (h)	7	14	21	28	35	42	49
**14**	**0,034**						
**21**	**0,001**	1,000					
**28**	0,259	**0,000**	**0,000**				
**35**	**0,000**	**0,000**	**0,000**	0,292			
**42**	0,259	**0,000**	**0,000**	1,000	0,417		
**49**	**0,001**	**0,000**	**0,000**	0,834	1,000	0,945	
**56**	**0,000**	**0,000**	**0,000**	0,307	1,000	0,435	1,000

***In bold p≤ 0.05**. Approximate Probabilities for Post Hoc Tests

Table S17: Tukey HSD with Benjamini-Hochberg correction for bioluminiscence in *Bmal1 -/-* cells (see Fig.6, Table 1)

Time (h)	7	14	21	28	35	42	49
**14**	1,000						
**21**	1,000	1,000					
**28**	0,327	0,218	0,153				
**35**	0,958	0,907	0,802	0,776			
**42**	0,999	0,997	0,979	0,487	0,999		
**49**	1,000	1,000	0,996	0,373	0,989	1,000	
**56**	1,000	1,000	1,000	0,180	0,854	0,990	0,999

***In bold p≤ 0.05**. Approximate Probabilities for Post Hoc Tests

Table S18: Tukey HSD with Benjamini-Hochberg correction for bioluminiscence in *wt* cells (see Fig.6, Table 1)

Time (h)	7	14	21	28	35	42	49
**14**	**0,001**						
**21**	**0,000**	0,983					
**28**	0,060	0,551	0,158				
**35**	**0,006**	0,995	0,735	0,920			
**42**	**0,000**	0,990	1,000	0,181	0,779		
**49**	**0,000**	0,994	1,000	0,199	0,809	1,000	
**56**	**0,001**	1,000	0,996	0,434	0,977	0,998	0,999

***In bold p≤ 0.05**. Approximate Probabilities for Post Hoc Tests
